# Supplementary material for: Difference in the recruitment of intrinsic foot muscles in the elderly under static and dynamic postural conditions
Source: PeerJ. 2023 Jul 19;11:e15719. doi: 10.7717/peerj.15719 (PMC10362842; doi:10.7717/peerj.15719)
Supplement: Supplemental Information 3 [file peerj-11-15719-s003.docx]

The document with file name “Muscle activation and CoP in the condictions of LOS test”, is the data of the LOS test in which the foot muscles’ activation magnitude was compared with the corresponding CoP parameter. Among these conditions, 41 represents the condition of forward-side task, 43 represents the condition of dominant-side task, 45 represents the condition of backward -side task, and the 47 represents the condition of non-dominant-side task.

In addition, the document named “Muscle activation of foot and ankle muscles in the conditions of SOT test”, is the activation of foot and ankle muscles in the six conditions in SOT test. All muscles’ activation is represented as as normalized percentage values (NRMS). TA, Tibialis anterior; LG,

Lateral head of gastrocnemius; MG, Medial head of gastrocnemius; PL, Peroneus longus; AbH, Abductor hallucis; FDB, Flexor digitorum brevis.
